# Molecular Virology of KSHV in the Lymphocyte Compartment—Insights From Patient Samples and *De Novo* Infection Models

**DOI:** 10.3389/fcimb.2020.607663

**Published:** 2020-12-04

**Authors:** Farizeh Aalam, Jennifer Totonchy

**Affiliations:** Biomedical and Pharmaceutical Sciences, Chapman University School of Pharmacy, Irvine, CA, United States

**Keywords:** KSHV, HHV8 (KSHV), virus-host interaction, B lymphcytes, immune evasion, hematological malignancies

## Abstract

The incidence of Kaposi’s sarcoma-associated herpesvirus (KSHV)-associated Kaposi Sarcoma has declined precipitously in the present era of effective HIV treatment. However, KSHV-associated lymphoproliferative disorders although rare, have not seen a similar decline. Lymphoma is now a leading cause of death in people living with HIV (PLWH), indicating that the immune reconstitution provided by antiretroviral therapy is not sufficient to fully correct the lymphomagenic immune dysregulation perpetrated by HIV infection. As such, novel insights into the mechanisms of KSHV-mediated pathogenesis in the immune compartment are urgently needed in order to develop novel therapeutics aimed at prevention and treatment of KSHV-associated lymphoproliferations. In this review, we will discuss our current understanding of KSHV molecular virology in the lymphocyte compartment, concentrating on studies which explore mechanisms unique to infection in B lymphocytes.

## Introduction

KSHV (HHV8) belongs to the gamma-herpesvirus family and is associated with both lymphoid and non-lymphoid cell tumors in humans (Chang et al., 1994). KSHV-associated malignancies occur primarily in the context of immunodeficiency. KSHV is the etiologic agent of Kaposi’s sarcoma, as well as the B cell lymphoproliferative disorders, primary effusion lymphoma (PEL), and multicentric castleman disease (MCD) (Chang et al., 1994; Cesarman et al., 1995) as well as the recently discovered KSHV inflammatory cytokine syndrome (KICS) (Uldrick et al., 2010).

**Figure 1:**
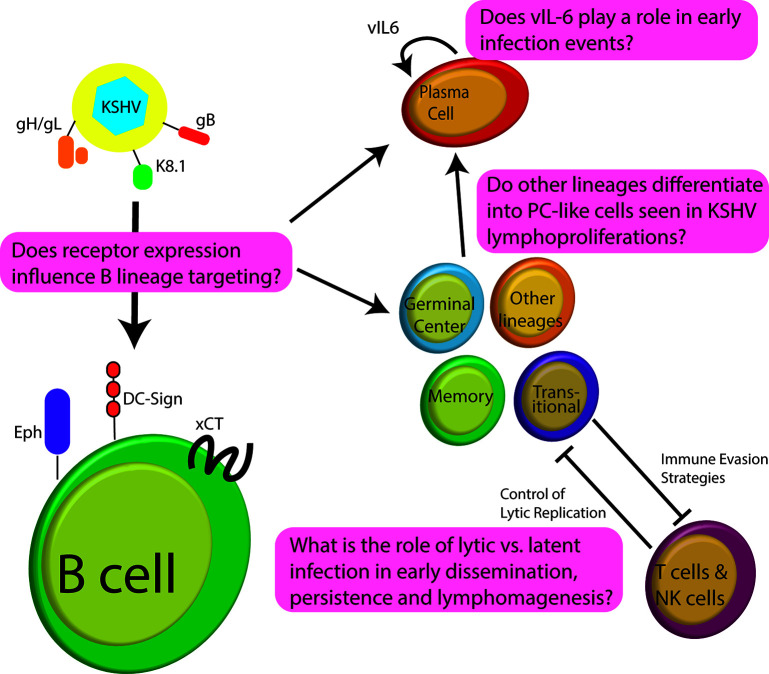
Schematic of early infection events for KSHV in B lymphocytes highlighting some of the significant questions that remain unanswered in the field.

Despite nearly three decades of research, not much is known regarding the early stages of development for KSHV lymphoproliferative disorders and the person-to-person transmission of KSHV. This can be partly explained by the host range limitation and broad *in vitro* cellular tropism of KSHV (Blackbourn et al., 2000; Bechtel et al., 2003). During the latent phase of infection, viral gene expression is limited and KSHV is maintained as an extrachromosomal episome; persisting for the lifetime of the individual  (Ueda, 2018). Like other herpesviruses, KSHV can become lytic under some physiological conditions (Grundhoff and Ganem, 2004; Li et al., 2014; Johnston et al., 2019; Wei et al., 2019). The process by which the lytic switch occurs and the relative contributions of lytic/latent phases to KSHV persistence are poorly understood. This is partly because expression and activity of the KSHV regulatory proteins appear to be cell type and tissue-specific and *in vivo* niches for persistence in humans remain poorly characterized (Rivas et al., 2001; Koch et al., 2019). There are significant gaps in our understanding of how KSHV targets B cells for infection and how the virus manipulates B cell physiology in the development of PEL and MCD (**Figure 1**). Further study of KSHV molecular virology in the lymphocyte compartment is needed to understand the pathogenesis of KSHV-associated lymphoproliferation so that effective treatment paradigms can be developed. In this review, we explore our current understanding of KSHV biology in B cells concentrating on studies which use *de novo* infection of human B cells, analysis of patient samples from KSHV lymphoproliferative disease, and relevant lymphoma cell lines. We have intentionally omitted discussion of KSHV manipulation of cytokine expression and signaling from this work as it is complex, and we have recently reviewed the topic comprehensively elsewhere (Alomari and Totonchy, 2020). Moreover, we have omitted discussion of humanized mouse models for KSHV infection as they have also been reviewed very recently (Münz, 2020).

## B Lymphocyte-Specific Molecular Virology of Kaposi’s Sarcoma-Associated Herpesvirus

### Entry Into B Cells

HHV-8 DNA is detectable in the B cells from both HIV+ and HIV-PEL and MCD cases (Dupin et al., 1999). Interestingly, KSHV isolated from EBV+PEL cells is able to infect B cells from seronegative patients (Mesri et al., 1996). Phylogenetic analysis and the association of KSHV infection with pathological lymphoproliferations are sufficient to characterize KSHV as a lymphotropic gamma-herpesvirus. However, primary B cells and B lymphoma cell lines show poor susceptibility to KSHV infection *in vitro* compared to adherent cell lines (Bechtel et al., 2003). The extensive *in vitro* susceptibility of adherent cell lines can partly be explained by the presence of various cellular receptors used by the viral glycoproteins for attachment and entry (Akula et al., 2001a; Akula et al., 2001b; Akula et al., 2002; Rappocciolo et al., 2008; Hahn et al., 2009; Chen et al., 2019; Großkopf et al., 2019; Muniraju et al., 2019)

KSHV virion attachment to adherent cells can be facilitated through heparan sulfate proteoglycans on the host cell surface (Akula et al., 2001a), and the low *in vitro* susceptibility of B cells has been attributed to a lack of HS expression. This theory is supported by the observation that restoration of cell surface HS in B cell lines results in increased susceptibility to infection (Jarousse et al., 2008; Jarousse et al., 2011; Dollery et al., 2019). The lectin DC-SIGN has also been implicated as an attachment factor for KSHV entry into B cells. Approximately, 8% of CD19+CD20+ peripheral blood B cells and 26% of tonsillar B cells are positive for DC-SIGN, and activation of peripheral blood B cells with IL-4 and CD40L results in 3 to 3.5 fold increase in DC-SIGN and CD23 expression (Rappocciolo et al., 2006). Activated B cells are more susceptible to KSHV infection and KSHV infected B cells show increased DC-SIGN levels compared to uninfected cells (Rappocciolo et al., 2008). Interestingly, B cells expressing DC-SIGN can bind and transfer HIV-1 virions to T cells (Rappocciolo et al., 2006). Taken together, these observations suggest that KSHV and HIV infections act synergistically. KSHV infection of B cells can facilitate the dissemination of HIV-1 to CD4+ T cells *via* upregulation of B cell DC-SIGN expression, and HIV, in turn, depletes the CD4+ T cell pool creating an immunological milieu in which KSHV benefits from the lack of immune surveillance.

KSHV encodes a variety of glycoproteins which facilitate virion attachment, fusion, and viral entry into the host cell. Among the various KSHV glycoproteins, gH/gL complex is proved to be the major antigenic determinant of KSHV-specific nAbs in the plasma of KS patients regardless of their disease status (Mortazavi et al., 2020), suggesting that this complex is critical for virus entry. Binding of gH/gL glycoprotein complex to the surface is not well characterized, but it is not HSPG-dependent (Hahn et al., 2009). KSHV entry into the BJAB cell line has been linked to gH/gL binding to EphA7 (Großkopf et al., 2019). Eph4 also binds to gH/gL, and is expressed in B cells, endothelial, fibroblast, and epithelial cells (Chen et al., 2019). In HEK293T cells, Eph4 binds more tightly with gH/gL than Eph2 (Chen et al., 2019). RNA sequencing data shows that B cells express Eph4 on their cell surface, albeit not as abundant as endothelial cells but higher than epithelial cells (Chen et al., 2019). Thus, it is possible that gH/gL complex can establish interaction with Eph4 in B cells, since B cells may have almost the same level of Eph4 as HEK293 (epithelial cells) on their surface. However, use of Eph4 as a KSHV entry receptor for B cells has not been studied specifically. Interestingly, the MC116 lymphoma cell line expresses both EphA7 and Eph4, and is susceptible to KSHV infection, but studies with a KSHV mutant lacking gH demonstrated that KSHV entry into MC116 cells is not dependent upon gH/gL (Muniraju et al., 2019). This study, in particular, highlights the significant gaps in our understanding of the molecular virology of KSHV entry into B cells.

Another study showed that K8.1A is required for KSHV infection of both MC116 and CD20+CD3− B cells from tonsil. The cellular receptor interacting with K8.1A in this context is not known, but it is independent of HS binding (Dollery et al., 2019). Finally, the KSHV glycoprotein gB, which is presumed to be the KSHV fusion protein, binds to DC-SIGN *in situ* in dose dependent manner (Hensler et al., 2014), but whether this interaction is essential for KSHV entry into B cells has not been formally studied. xCT, the light chain has been shown to be involved in KSHV fusion and entry in several cell lines. Although, its mRNA expression is undetectable in CD19+ PBMCs (Kaleeba and Berger, 2006a; Kaleeba and Berger, 2006b) xCT is highly expressed on the surface of PEL cell lines and targeting it by xCT selective inhibitor, induces apoptosis in caspase dependent manner. Selective inhibition of xCT in immune deficient mouse xenograft model proves that it plays key role in tumor progression, survival, and growth of PEL cells (Dai et al., 2014). The expression of xCT can be induced by KSHV miRNAs conferring permissiveness to KSHV in murine microphages and HUVEC cells. Additionally, the expression of xCT within the KS lesion is correlated with the tumor stage (Qin et al., 2010).Whether KSHV miRNAs and change in redox balance contribute to upregulation of xCT in primary B cells to increase the KSHV permissiveness, remains to be answered.

To date, no comprehensive studies been done on primary human B cells samples to elucidate the cellular receptors involved in KSHV entry into B lymphocytes or the individual and collective contributions of KSHV glycoproteins to this process. Further studies are needed to determine these important interactions to facilitate the rational design of vaccine strategies that will effectively limit the establishment of infection in the lymphocyte compartment.

### Manipulation of the Cell Cycle

KSHV can establish latent infection in many adherent cell lines, including human and non-human cells of epithelial, endothelial, and mesenchymal origin (Bechtel et al., 2003). Previous studies in primary human B cells report that infection is lytic, particularly in the absence of T cells, but what controls the lytic switch in these cells remains to be established (Myoung and Ganem, 2011a). In addition to T cell control of latency, B cell immunophenotype and activation state have been implicated as factors influencing the lytic/latent balance in B cells (Rappocciolo et al., 2008; Hassman et al., 2011; Myoung and Ganem, 2011a), as well as the immunological status of the individual and the presence of other pathogens (Gregory et al., 2009).

Although the latent phase of infection allows viral persistence and immune-evasion, the production of viral progeny and viral transmission and spread between the cells, depends on the lytic phase. *De novo* infected PBMCs exhibit simultaneous expression of numerous latent and lytic markers at the very beginning of the infection (Purushothaman et al., 2015). This short lytic replication seems to be a prerequisite for the establishment of the latent phase in PBMCs infected with EBV (Halder et al., 2009). Nevertheless, the lytic gene expression is not required for KSHV infection of PBMCs before or after EBV infection or mitogenic activation (Faure et al., 2019). Do B cells represent a significant source of KSHV virions during human infection? The early lytic gene K8 (K-bZIP), a cell cycle regulator showing homology to EBV’ Zta, is required for viral lytic DNA replication and virion production in PEL cell lines (Wu et al., 2002; Lefort and Flamand, 2009). Its expression concurs with augmented C/EBPα, p21 and p27 in the nucleus, causing the cell arrest in G1 phase (Wu et al., 2002; Izumiya et al., 2003a). This prolonged G1 arrest is as a result of K8 binding to CKD2, interfering its kinase activity, giving ample time for viral early gene transcription and translation (Izumiya et al., 2003a). K8 also interacts with p53 inhibiting its transcription, preventing apoptosis (Park et al., 2000). However, in another study by Hollingworth et al., lytic replication in PEL cells was shown to require S phase entry (Hollingworth et al., 2020). Replication and transcription activator (RTA) is a protein encoded by ORF8 has been shown to co-localize with K8 within the nucleus of the PEL cells, and its association with the K8 (Izumiya et al., 2003b) can initiate lytic reactivation from latency by binding to a particular sequence on the host and viral DNA further modulating the transcription of viral and host regulatory genes throughout KSHV lytic reactivation (Kaul et al., 2019). Viral DNA replication is controlled by both transcriptional coactivator p300 and CBP. P300 was shown to be involved in the oncogenesis of PEL by driving B cell proliferation and inhibiting KSHV lytic replication. Knockout of p300 in PEL cells decreased KSHV genome copy number and virion production by suppressing lytic gene expression, possibly maintaining the latency of KSHV *via* binding with ATF3 (Sun et al., 2020). Nonsense-mediated mRNA decay (NMD) is an RNA quality control implemented by the cells to restrict the action of the RNA viruses and serve cellular quality control. Interestingly, viral RTA’ mRNA is targeted by NMD, impeding KSHV lytic reactivation in PEL cells (Zhao et al., 2020). However, KSHV has evolved to overcome some of these quality controls and exonuclease activities by circularizing its structural and regulatory RNAs incorporated into the virions (Abere et al., 2020). Taken together, the current literature demonstrates that multiple layers of both viral and cellular regulation influence KSHV latency and lytic reactivation in B cells. It is notable that most of this work has been done in PEL cells, and future studies investigating how KSHV manipulates the cell cycle and cell type specific control of latency and reactivation in primary B lymphocytes will be critical for understanding early events in KSHV infection and pathogenesis of KSHV-associated lymphoproliferative diseases.

### Kaposi’s Sarcoma-Associated Herpesvirus Immune Evasion

KSHV infection persists for the lifetime of the host and, like all herpesviruses, KSHV must have an arsenal of mechanisms for evading host immunity in order to accomplish this. Lymphotropic gamma-herpesviruses are particularly interesting in this regard because they can manipulate and evade the host immune system *via* mechanisms that require direct infection of immune cells. Moreover, the inflammatory nature of KSHV-associated malignancies indicates that KSHV immune-evasion mechanisms may also directly contribute to pathogenesis in KSHV-associated diseases. Indeed, this immune-evasion is manifested at the transcriptional level within the first few hours of infection, by hampering the expression of immune response genes and inducing the proapoptotic regulators in BJAB cells (Naranatt et al., 2004). KSHV can infect both B and T cells in tonsil primary cell culture, however evidence suggests that infection of T cells is abortive (Myoung and Ganem, 2011b). Moreover, there is reciprocal activation of T cells by KSHV-infected B cells and contact-dependent control of KSHV lytic reactivation by T cells in *ex vivo* tonsil cultures, and in this system the activation of T cells is independent of both KSHV antigen and MHC restriction (Myoung and Ganem, 2011a).

Activated, KSHV infected B lymphocytes from PBMC and tonsils show downregulation of MHC class I (HLA-A, HLA-B, and HLA-C) within 24 h of infection as well as decreased expression of CD20 (Rappocciolo et al., 2008). Modulation of MHC class I expression is also observed in PEL derived B cell-lines and is thought to be partially due to reduced expression of the TAP-1 gene. Importantly, this MHC-I modulation can disrupt cytotoxic T lymphocyte surveillance of KSHV infected cells (Brander et al., 2000), aiding in KSHV persistence and tumorigenesis in B cells. The CD20 low phenotype of KSHV infected cells is also present in MCD and may limit B cell-targeted treatment options for MCD patients. However, these patients still show clinical benefit from rituximab (an anti-CD20 monoclonal antibody) treatment (Naresh et al., 2009).

KSHV encodes four viral interferon regulatory factors (vIRFs). These proteins have minimal homology to human IRFs, but vIRF1, vIRF2, and vIRF3 are known to bind DNA elements similar to their human IRF counterparts (Lubyova and Pitha, 2000; Park et al., 2007; Hu et al., 2016) vIRFs exert their regulatory role at varying levels ranging from hampering the antiviral interferon response to inhibition of signaling pathways to control the function of cellular proteins, thereby interfering with the cellular processes such as apoptosis (Rivas et al., 2001; Nakamura et al., 2001; Lee et al., 2009) proliferation and angiogenesis (Wies et al., 2008; Li et al., 2019; Li et al., 2020) vIRF3 (LANA-2) expression is detected in nearly all virus infected cells in PEL and MCD tumors, and vIRF3 is a bona fide oncogene which can inhibit the function of p53. Moreover, among the KSHV vIRFs, the function of vIRF3 is thought to be B cell-specific. Interestingly, the expression level of vIRF3 does not fluctuate even after lytic reactivation (Rivas et al., 2001), suggesting that there is an alternative level of regulation driving vIRF3 expression in B cells. In latently infected PEL cell lines, vIRF3 is linked to decreased MHC-II expression, and vIRF3 also modulates both type II (Schmidt et al., 2011) and type I interferon responses (Lubyova et al., 2004). vIRF3-mediated inhibiton of IFN*γ* results in inhibition of both PIII and PIV promoter of class II transactivator (CIITA) transcription (Schmidt et al., 2011). Importantly, vIRF3 is required for the survival of both EBV co-infected and EBV negative B cell lymphomas *in vitro* (Wies et al., 2008).

### Kaposi’s Sarcoma-Associated Herpesvirus Modulation of B Cell Phenotypes

#### The Proliferation and Plasmablast Differentiation

PEL is an immunoblastic tumor affecting the pericardial or pleural area of the body cavities. PEL tumor cells are negative for most B cell surface markers except CD138/syndecan, a marker of terminal plasma cell differentiation (Jenner et al., 2003). These terminally differentiated CD138+CD20+ and CD20− plasma cells are highly targeted by KSHV infection in primary B cells of tonsillar sample, gaining greater survival rate for CD20− cells over 3 days post infection. This indirect survival effect is as a result of differentiation of other B cell lineages into the CD138+ cells (Aalam et al., 2020). Interestingly, more than 60% of the KSHV infected B cells from PBMCs of KS positive patients are positive for CD138 (Bella et al., 2010). In MCD, the pathological cells are monotypic/polyclonal plasmablasts located in the mantle zone of spleen and lymph nodes (Du et al., 2001). These cells express PRDM1 / BLIMP1 marking them as pre-plasma or terminal plasma stage of B-cell differentiation (Chadburn et al., 2008). Most of KSHV infected B cells in MCD patients express IL-6 (Du et al., 2001), and the importance of IL-6 signaling in MCD is illustrated by the finding that tocilizumab (an IL-6R blocking monoclonal antibody) can ameliorate the symptoms or even lead to prolonged remission in some MCD cases (Song et al., 2010; Galeotti et al., 2012; Ramaswami et al., 2020). In *ex vivo* infection models, particularly those performed in tonsillar B lymphocytes, the immunophenotype of infected cells closely resembles the pathological cells present in MCD (Du et al., 2001; Chadburn et al., 2008; Totonchy et al., 2018). Latently KSHV infected B cells from the tonsil (characterized by LANA dots), proliferate, and express a high level of IL-6R and CD27 on their surface exhibiting plasma blast morphology at 72 h post-infection (Hassman et al., 2011). Similarly, KSHV infection of naïve B lymphocytes from human tonsil upregulates IL-6 secretion as well as CD27 expression (Totonchy et al., 2018). *Ex vivo* infection of activated peripheral blood B cells expressing DC-SIGN results in infection of primarily naive and IgM memory B cells at early times post-infection (Rappocciolo et al., 2008). Remarkably, a similar expansion of MZ-like memory and naive B cells is seen in PBMC from HIV negative KS patients (Bella et al., 2010). Taken together, the concordance between pre-disease immunophenotypes, *ex vivo* infection immunophenotypes and the phenotypes seen in KSHV lymphoproliferative diseases suggests that KSHV infection manipulates the B cell compartment toward particular immunophenotypes even in the absence of overt KSHV-associated lymphoproliferation.

#### Induction of Immunoglobulin Light Chain Revision

One of the more puzzling characteristics of MCD is the fact that KSHV infection is restricted to Igλ positive B lymphocytes in patient samples (Du et al., 2001). The same restriction is observed in KSHV infected B lymphocytes derived from tonsil samples (Hassman et al., 2011). Moreover, in PEL, most infected B cells are lg negative with occasional Igλ positive B cells (Matolcsy et al., 1998). Our group was able to show that KSHV infection in Igκ tonsil lymphocytes induces Igλ expression *via* re-induction of V(D)J recombination driving BCR revision. These cells express LANA, K8.1 and ORF59 markers, indicating a mixed population of lymphocytes in latent and lytic stages of infection (Totonchy et al., 2018). The same study also detects the Igλ+ KSHV infected cells in biopsies of HIV positive patients with AIDS-related lymphadenopathy (ARL) having no histologically similar characteristics of MCD, again supporting the conclusion that KSHV manipulates B cell physiology even in the absence of KSHV-associated lymphoproliferative disease and establishing that the Igλ+ phenotype in MCD is driven directly by KSHV infection. Further study is needed to characterize the intervening events that drive KSHV infected B cells from these early manipulations of B cell phenotype and physiology to overt pathological lymphoproliferation.

## Discussion

KSHV has been co-evolving within the human immune system for thousands of years, and it has developed a plethora of mechanisms for manipulating both B cell physiology and overall immunology which are just beginning to be understood. In recent years, progress on this has been accelerated by new models allowing efficient infection of tonsil-derived primary B cells (Kang and Myoung, 2017). Some of these primary human samples can last up to 10 days, giving ample time to explore the early infection events. One of the hurdle of studying human primary B cell is their limited survival and difficulty of immortalizing them. In the study by Faure et al. (2019), they could achieve up to 20 fold increase in KSHV infection of peripheral B cells co-infected with EBV. These cells were best infected when exposed to KSHV within 24 h of EBV infection and could survive for months under culture conditions. In our recent paper (Aalam et al., 2020), we have generated a library of 40 tonsil specimen and included detailed B cell subtype analysis and *de novo* infection model. The samples exhibited diverse range of susceptibilities and determined varieties of B cells are susceptible to KSHV infection with CD138+ cells being highly targeted population. However, detailed infection analysis on what drives the susceptibility on these samples are missing as is information about the contributions of cellular and viral genes as well as the immunological milieu to the emergence of pathological lymphoproliferation. While, adherent cells and B cell lines are extensively used for their convenience and ease of manipulation, confirming the same findings within the human primary cells should not be overlooked. Therefore, more systematic and detailed studies are required to evaluate KSHV molecular virology in primary B cells to decode the dynamics of KSHV pathology in the lymphocyte compartment.

## Author Contributions

FA was responsible for content curation and writing of the manuscript. JT was responsible for editing the manuscript, supervision, and obtaining funding. All authors contributed to the article and approved the submitted version.

## Funding

This work was funded by NIH National Cancer Institute, grant number R01CA239590.

## Conflict of Interest

The authors declare that the research was conducted in the absence of any commercial or financial relationships that could be construed as a potential conflict of interest.

## References

[B1] Aalam F. Nabiee R. Castano J. R. Totonchy J. (2020). Analysis of KSHV B Lymphocyte Lineage Tropism in Human Tonsil Reveals Efficient Infection of CD138+ Plasma Cells. PloS Pathog. 16 (10), e1008968. 10.1371/journal.ppat.1008968 33075105 PMC7595638

[B2] Abere B. Li J. Zhou H. Toptan T. Moore P. S. Chang Y. (2020). Kaposi’s Sarcoma-Associated Herpesvirus-Encoded CircRNAs Are Expressed in Infected Tumor Tissues and Are Incorporated into Virions. MBio 11 (1), e03027–19. 10.1128/mBio.03027-19 PMC6946807 31911496

[B3] Akula S. M. Pramod N. P. Wang F. Z. Chandran B. (2001a). Human Herpesvirus 8 Envelope-Associated Glycoprotein B Interacts with Heparan Sulfate-like Moieties. Virology 284 (2), 235–249. 10.1006/viro.2001.0921 11384223

[B4] Akula S. M. Wang F. Z. Vieira J. Chandran B. (2001b). Human Herpesvirus 8 Interaction with Target Cells Involves Heparan Sulfate. Virology 282 (2), 245–255. 10.1006/viro.2000.0851 11289807

[B5] Akula S. M. Pramod N. P. Wang F. Z. Chandran B. (2002). Integrin Alpha3beta1 (CD 49c/29) Is a Cellular Receptor for Kaposi’s Sarcoma-Associated Herpesvirus (KSHV/HHV-8) Entry into the Target Cells. Cell 108 (3), 407–419. 10.1016/s0092-8674(02)00628-1 11853674

[B6] Alomari N. Totonchy J. (2020). Cytokine-Targeted Therapeutics for KSHV-Associated Disease. Viruses 12 (10), 1097. 10.3390/v12101097 PMC7600567 32998419

[B7] Bechtel J. T. Liang Y. Hvidding J. Ganem D. (2003). Host Range of Kaposi’s Sarcoma-Associated Herpesvirus in Cultured Cells. J. Virol. 77 (11), 6474–6481. 10.1128/jvi.77.11.6474-6481.2003 12743304 PMC155009

[B8] Bella S. D. Taddeo A. Colombo E. Brambilla L. Bellinvia M. Pregliasco F. (2010). Human Herpesvirus-8 Infection Leads to Expansion of the Preimmune/Natural Effector B Cell Compartment. PloS One 5 (11), e15029. 10.1371/journal.pone.0015029 21124778 PMC2993943

[B9] Blackbourn D. J. Lennette E. Klencke B. Moses A. Chandran B. Weinstein M. (2000). The Restricted Cellular Host Range of Human Herpesvirus 8. AIDS (London England) 14 (9), 1123–1133. 10.1097/00002030-200006160-00009 10894276

[B10] Brander C. Suscovich T. Lee Y. Nguyen P. T. O’Connor P. Seebach J. (2000). Impaired CTL Recognition of Cells Latently Infected with Kaposi’s Sarcoma-Associated Herpes Virus. J. Immunol. (Baltimore Md. : 1950) 165 (4), 2077–2083. 10.4049/jimmunol.165.4.2077 10925292

[B11] Cesarman E. Chang Y. Moore P. S. Said J. W. Knowles D. M. (1995). Kaposi’s Sarcoma-Associated Herpesvirus-like DNA Sequences in AIDS-Related Body-Cavity-Based Lymphomas. New Engl. J. Med. 332 (18), 1186–1191. 10.1056/NEJM199505043321802 7700311

[B12] Chadburn A. Hyjek E. M. Tam W. Liu Y. Rengifo T. Cesarman E. (2008). Immunophenotypic Analysis of the Kaposi Sarcoma Herpesvirus (KSHV; HHV-8)-Infected B Cells in HIV+ Multicentric Castleman Disease (MCD). Histopathology 53 (5), 513–524. 10.1111/j.1365-2559.2008.03144.x 18983461

[B13] Chang Y. Cesarman E. Pessin M. S. Lee F. Culpepper J. Knowles D. M. (1994). Identification of Herpesvirus-like DNA Sequences in AIDS-Associated Kaposi’s Sarcoma. Science (New York N.Y.) 266 (5192), 1865–1869. 10.1126/science.7997879 7997879

[B14] Chen J. Zhang X. Schaller S. Jardetzky T. S. Longnecker R. (2019). Ephrin Receptor A4 Is a New Kaposi’s Sarcoma-Associated Herpesvirus Virus Entry Receptor. MBio 10 (1), e02892–18. 10.1128/mBio.02892-18 30782663 PMC6381284

[B15] Dai L. Cao Y. Chen Y. Parsons C. Qin Z. (2014). Targeting XCT, a Cystine-Glutamate Transporter Induces Apoptosis and Tumor Regression for KSHV/HIV-Associated Lymphoma. J. Hematol. Oncol. 7 (April):30. 10.1186/1756-8722-7-30 24708874 PMC4234972

[B16] Dollery S. J. Santiago-Crespo R. J. Chatterjee D. Berger E. A. (2019). Glycoprotein K8.1A of Kaposi’s Sarcoma-Associated Herpesvirus Is a Critical B Cell Tropism Determinant Independent of Its Heparan Sulfate Binding Activity. J. Virol. 93 (6), e01876–18. 10.1128/JVI.01876-18 30567992 PMC6401448

[B17] Du M. Q. Liu H. Diss T. C. Ye H. Hamoudi R. A. Dupin N. (2001). Kaposi Sarcoma-Associated Herpesvirus Infects Monotypic (IgM Lambda) but Polyclonal Naive B Cells in Castleman Disease and Associated Lymphoproliferative Disorders. Blood 97 (7), 2130–2136. 10.1182/blood.v97.7.2130 11264181

[B18] Dupin N. Fisher C. Kellam P. Ariad S. Tulliez M. Franck N. (1999). Distribution of Human Herpesvirus-8 Latently Infected Cells in Kaposi’s Sarcoma, Multicentric Castleman’s Disease, and Primary Effusion Lymphoma. Proc. Natl. Acad. Sci. U.S.A. 96 (8), 4546–4551. 10.1073/pnas.96.8.4546 10200299 PMC16369

[B19] Faure A. Hayes M. Sugden B. (2019). How Kaposi’s Sarcoma-Associated Herpesvirus Stably Transforms Peripheral B Cells towards Lymphomagenesis. Proc. Natl. Acad. Sci. U.S.A. 116 (33), 16519–16528. 10.1073/pnas.1905025116 31363046 PMC6697783

[B20] Galeotti C. Boucheron A. Guillaume S. Koné-Paut I. (2012). Sustained Remission of Multicentric Castleman Disease in Children Treated with Tocilizumab, an Anti-Interleukin-6 Receptor Antibody. Mol. Cancer Ther. 11 (8), 1623–1626. 10.1158/1535-7163.MCT-11-0972 22638145

[B21] Gregory S. M. West J. A. Dillon P. J. Hilscher C. Dittmer D. P. Damania B. (2009). Toll-like Receptor Signaling Controls Reactivation of KSHV from Latency. Proc. Natl. Acad. Sci. U.S.A. 106 (28), 11725–11730. 10.1073/pnas.0905316106 19564611 PMC2710638

[B22] Großkopf A. K. Schlagowski S. Hörnich B. F. Fricke T. Desrosiers R. C. Hahn A. S. (2019). EphA7 Functions as Receptor on BJAB Cells for Cell-to-Cell Transmission of the Kaposi’s Sarcoma-Associated Herpesvirus and for Cell-Free Infection by the Related Rhesus Monkey Rhadinovirus. J. Virol. 93 (15), e00064–19. 10.1128/JVI.00064-19 31118261 PMC6639272

[B23] Grundhoff A. Ganem D. (2004). Inefficient Establishment of KSHV Latency Suggests an Additional Role for Continued Lytic Replication in Kaposi Sarcoma Pathogenesis. J. Clin. Invest. 113 (1), 124–136. 10.1172/JCI17803 14702116 PMC300762

[B24] Hahn A. Birkmann A. Wies E. Dorer D. Mahr K. Stürzl M. (2009). Kaposi’s Sarcoma-Associated Herpesvirus GH/GL: Glycoprotein Export and Interaction with Cellular Receptors. J. Virol. 83 (1), 396–407. 10.1128/JVI.01170-08 18945775 PMC2612313

[B25] Halder S. Murakami M. Verma S. C. Kumar P. Yi F. Robertson E. S. (2009). Early Events Associated with Infection of Epstein-Barr Virus Infection of Primary B-Cells. PloS One 4 (9), e7214. 10.1371/journal.pone.0007214 19784370 PMC2746279

[B26] Hassman L. M. Ellison T. J. Kedes D. H. (2011). KSHV Infects a Subset of Human Tonsillar B Cells, Driving Proliferation and Plasmablast Differentiation. J. Clin. Invest. 121 (2), 752–768. 10.1172/JCI44185 21245574 PMC3026728

[B27] Hensler H. R. Tomaszewski M. J. Rappocciolo G. Rinaldo C. R. Jenkins F. J. (2014). Human Herpesvirus 8 Glycoprotein B Binds the Entry Receptor DC-SIGN. Virus Res. 190 (September), 97–103. 10.1016/j.virusres.2014.07.003 25018023 PMC4142096

[B28] Hollingworth R. Stewart G. S. Grand R. J. (2020). Productive Herpesvirus Lytic Replication in Primary Effusion Lymphoma Cells Requires S-Phase Entry. J. Gen. Virol. 101 (8), 873–883. 10.1099/jgv.0.001444 32501196 PMC7641394

[B29] Hu H. Dong J. Liang D. Gao Z. Bai L. Sun R. (2016). Genome-Wide Mapping of the Binding Sites and Structural Analysis of Kaposi’s Sarcoma-Associated Herpesvirus Viral Interferon Regulatory Factor 2 Reveal That It Is a DNA-Binding Transcription Factor. J. Virol. 90 (3), 1158–1168. 10.1128/JVI.01392-15 26537687 PMC4719618

[B30] Izumiya Y. Lin S.-F. Ellison T. Chen L.-Y. Izumiya C. Luciw P. (2003a). Kaposi’s Sarcoma-Associated Herpesvirus K-BZIP Is a Coregulator of K-Rta: Physical Association and Promoter-Dependent Transcriptional Repression. J. Virol. 77 (2), 1441–1451. 10.1128/jvi.77.2.1441-1451.2003 12502859 PMC140808

[B31] Izumiya Y. Lin S.-F. Ellison T. J. Levy A. M. Mayeur G. L. Izumiya C. (2003b). Cell Cycle Regulation by Kaposi’s Sarcoma-Associated Herpesvirus K-BZIP: Direct Interaction with Cyclin-CDK2 and Induction of G1 Growth Arrest. J. Virol. 77 (17), 9652–9661. 10.1128/jvi.77.17.9652-9661.2003 12915577 PMC187423

[B32] Jarousse N. Chandran B. Coscoy L. (2008). Lack of Heparan Sulfate Expression in B-Cell Lines: Implications for Kaposi’s Sarcoma-Associated Herpesvirus and Murine Gammaherpesvirus 68 Infections. J. Virol. 82 (24), 12591–12597. 10.1128/JVI.01167-08 18842731 PMC2593311

[B33] Jarousse N. Trujillo D. L. Wilcox-Adelman S. Coscoy L. (2011). Virally-Induced Upregulation of Heparan Sulfate on B Cells via the Action of Type I IFN. J. Immunol. (Baltimore Md. : 1950) 187 (11), 5540–5547. 10.4049/jimmunol.1003495 PMC3677732 22048770

[B34] Jenner R. G. Maillard K. Cattini N. Weiss R. A. Boshoff C. Wooster R. (2003). Kaposi’s Sarcoma-Associated Herpesvirus-Infected Primary Effusion Lymphoma Has a Plasma Cell Gene Expression Profile. Proc. Natl. Acad. Sci. U.S.A. 100 (18), 10399–10404. 10.1073/pnas.1630810100 12925741 PMC193573

[B35] Johnston B. P. Pringle E. S. McCormick C. (2019). KSHV Activates Unfolded Protein Response Sensors but Suppresses Downstream Transcriptional Responses to Support Lytic Replication. PloS Pathog. 15 (12), e1008185. 10.1371/journal.ppat.1008185 31790507 PMC6907875

[B36] Kaleeba J. A.R. Berger E. A. (2006a). Kaposi’s Sarcoma-Associated Herpesvirus Fusion-Entry Receptor: Cystine Transporter XCT. Science (New York N.Y.) 311 (5769), 1921–1924. 10.1126/science.1120878 16574866

[B37] Kaleeba J. A.R. Berger E. A. (2006b). Broad Target Cell Selectivity of Kaposi’s Sarcoma-Associated Herpesvirus Glycoprotein-Mediated Cell Fusion and Virion Entry. Virology 354 (1), 7–14. 10.1016/j.virol.2006.06.009 16889811

[B38] Kang S. Myoung J. (2017). Primary Lymphocyte Infection Models for KSHV and Its Putative Tumorigenesis Mechanisms in B Cell Lymphomas. J. Microbiol. (Seoul Korea) 55 (5), 319–329. 10.1007/s12275-017-7075-2 28455586

[B39] Kaul R. Purushothaman P. Uppal T. Verma S. C. (2019). KSHV Lytic Proteins K-RTA and K8 Bind to Cellular and Viral Chromatin to Modulate Gene Expression. PloS One 14 (4), e0215394. 10.1371/journal.pone.0215394 30998737 PMC6472759

[B40] Koch S. Damas M. Freise A. Hage E. Dhingra A. Rückert J. (2019). Kaposi’s Sarcoma-Associated Herpesvirus VIRF2 Protein Utilizes an IFN-Dependent Pathway to Regulate Viral Early Gene Expression. PloS Pathog. 15 (5), e1007743. 10.1371/journal.ppat.1007743 31059555 PMC6522069

[B41] Lee H.-R. Toth Z. Shin Y. C. Lee J.-S. Chang H. Gu W. (2009). Kaposi’s Sarcoma-Associated Herpesvirus Viral Interferon Regulatory Factor 4 Targets MDM2 to Deregulate the P53 Tumor Suppressor Pathway. J. Virol. 83 (13), 6739–6747. 10.1128/JVI.02353-08 19369353 PMC2698538

[B42] Lefort S. Flamand L. (2009). Kaposi’s Sarcoma-Associated Herpesvirus K-BZIP Protein Is Necessary for Lytic Viral Gene Expression, DNA Replication, and Virion Production in Primary Effusion Lymphoma Cell Lines. J. Virol. 83 (11), 5869–5880. 10.1128/JVI.01821-08 19321621 PMC2681977

[B43] Li D.-J. Verma D. Mosbruger T. Swaminathan S. (2014). CTCF and Rad21 Act as Host Cell Restriction Factors for Kaposi’s Sarcoma-Associated Herpesvirus (KSHV) Lytic Replication by Modulating Viral Gene Transcription. PloS Pathog. 10 (1), e1003880. 10.1371/journal.ppat.1003880 24415941 PMC3887114

[B44] Li W. Wang Q. Feng Q. Wang F. Yan Q. Gao S.-J. (2019). Oncogenic KSHV-Encoded Interferon Regulatory Factor Upregulates HMGB2 and CMPK1 Expression to Promote Cell Invasion by Disrupting a Complex LncRNA-OIP5-AS1/MiR-218-5p Network. PloS Pathog. 15 (1), e1007578. 10.1371/journal.ppat.1007578 30699189 PMC6370251

[B45] Li W. Wang F. Shi J. Feng Q. Chen Y. Qi X. (2020). Sperm Associated Antigen 9 Promotes Oncogenic KSHV-Encoded Interferon Regulatory Factor-Induced Cellular Transformation and Angiogenesis by Activating the JNK/VEGFA Pathway. PloS Pathog. 16 (8), e1008730. 10.1371/journal.ppat.1008730 32776977 PMC7446834

[B46] Lubyova B. Pitha P. M. (2000). Characterization of a Novel Human Herpesvirus 8-Encoded Protein, VIRF-3, That Shows Homology to Viral and Cellular Interferon Regulatory Factors. J. Virol. 74 (17), 8194–8201. 10.1128/jvi.74.17.8194-8201.2000 10933732 PMC112355

[B47] Lubyova B. Kellum M. J. Frisancho A. J. Pitha P. M. (2004). Kaposi’s Sarcoma-Associated Herpesvirus-Encoded VIRF-3 Stimulates the Transcriptional Activity of Cellular IRF-3 and IRF-7. J. Biol. Chem. 279 (9), 7643–7654. 10.1074/jbc.M309485200 14668346

[B48] Matolcsy A. Nádor R. G. Cesarman E. Knowles D. M. (1998). Immunoglobulin VH Gene Mutational Analysis Suggests That Primary Effusion Lymphomas Derive from Different Stages of B Cell Maturation. Am. J. Pathol. 153 (5), 1609–1614. 10.1016/S0002-9440(10)65749-5 9811353 PMC1853415

[B49] Mesri E. A. Cesarman E. Arvanitakis L. Rafii S. Moore M. A. Posnett D. N. (1996). Human Herpesvirus-8/Kaposi’s Sarcoma-Associated Herpesvirus Is a New Transmissible Virus That Infects B Cells. J. Exp. Med. 183 (5), 2385–2390. 10.1084/jem.183.5.2385 8642350 PMC2192551

[B50] Mortazavi Y. Lidenge S. J. Tran T. West J. T. Wood C. Tso F. Y. (2020). The Kaposi’s Sarcoma-Associated Herpesvirus (KSHV) GH/GL Complex Is the Predominant Neutralizing Antigenic Determinant in KSHV-Infected Individuals. Viruses 12 (3), 256. 10.3390/v12030256 PMC7150787 32111001

[B51] Muniraju M. Mutsvunguma L. Z. Foley J. Escalante G. M. Rodriguez E. Nabiee R. (2019). Kaposi Sarcoma-Associated Herpesvirus Glycoprotein H Is Indispensable for Infection of Epithelial, Endothelial, and Fibroblast Cell Types. J. Virol. 93 (16), e00630–19. 10.1128/JVI.00630-19 31142670 PMC6675886

[B52] Münz C. (2020). Probing Reconstituted Human Immune Systems in Mice With Oncogenic γ-Herpesvirus Infections. Front. Immunol. 11:581419. 10.3389/fimmu.2020.581419 33013936 PMC7509489

[B53] Myoung J. Ganem D. (2011a). Active Lytic Infection of Human Primary Tonsillar B Cells by KSHV and Its Noncytolytic Control by Activated CD4+ T Cells. J. Clin. Invest. 121 (3), 1130–1140. 10.1172/JCI43755 21339648 PMC3049404

[B54] Myoung J. Ganem D. (2011b). Infection of Primary Human Tonsillar Lymphoid Cells by KSHV Reveals Frequent but Abortive Infection of T Cells. Virology 413 (1), 1–11. 10.1016/j.virol.2010.12.036 21353276 PMC3070441

[B55] Nakamura H. Li M. Zarycki J. Jung J. U. (2001). Inhibition of P53 Tumor Suppressor by Viral Interferon Regulatory Factor. J. Virol. 75 (16), 7572–7582. 10.1128/JVI.75.16.7572-7582.2001 11462029 PMC114992

[B56] Naranatt P. P. Krishnan H. H. Svojanovsky S. R. Bloomer C. Mathur S. Chandran B. (2004). Host Gene Induction and Transcriptional Reprogramming in Kaposi’s Sarcoma-Associated Herpesvirus (KSHV/HHV-8)-Infected Endothelial, Fibroblast, and B Cells: Insights into Modulation Events Early during Infection. Cancer Res. 64 (1), 72–84. 10.1158/0008-5472.can-03-2767 14729610

[B57] Naresh K. N. Trivedi P. Horncastle D. Bower M. (2009). CD20 Expression in the HHV-8-Infected Lymphoid Cells in Multicentric Castleman Disease. Histopathology Engl. 55 (3), 358–359. 10.1111/j.1365-2559.2009.03344.x 19723153

[B58] Park J. Seo T. Hwang S. Lee D. Gwack Y. Choe J. (2000). The K-BZIP Protein from Kaposi’s Sarcoma-Associated Herpesvirus Interacts with P53 and Represses Its Transcriptional Activity. J. Virol. 74 (24), 11977–11982. 10.1128/jvi.74.24.11977-11982.2000 11090200 PMC112483

[B59] Park J. Lee M.-S. Yoo S.-M. Jeong K. W. Lee D. Choe J. (2007). Identification of the DNA Sequence Interacting with Kaposi’s Sarcoma-Associated Herpesvirus Viral Interferon Regulatory Factor 1. J. Virol. 81 (22), 12680–12684. 10.1128/JVI.00556-07 17855527 PMC2169006

[B60] Purushothaman P. Thakker S. Verma S. C. (2015). Transcriptome Analysis of Kaposi’s Sarcoma-Associated Herpesvirus during de Novo Primary Infection of Human B and Endothelial Cells. J. Virol. 89 (6), 3093–3111. 10.1128/JVI.02507-14 25552714 PMC4337554

[B61] Qin Z. Freitas E. Sullivan R. Mohan S. Bacelieri R. Branch D. (2010). Upregulation of XCT by KSHV-Encoded MicroRNAs Facilitates KSHV Dissemination and Persistence in an Environment of Oxidative Stress. PloS Pathog. 6 (1), e1000742. 10.1371/journal.ppat.1000742 20126446 PMC2813276

[B62] Ramaswami R. Lurain K. Peer C. J. Serquiña A. Wang V. Widell A. (2020). Tocilizumab in Patients with Symptomatic Kaposi Sarcoma Herpesvirus-Associated Multicentric Castleman Disease. Blood 135 (25), 2316–2319. 10.1182/blood.2019004602 32276276 PMC7316216

[B63] Rappocciolo G. Piazza P. Fuller C. L. Reinhart T. A. Watkins S. C. Rowe D. T. (2006). DC-SIGN on B Lymphocytes Is Required for Transmission of HIV-1 to T Lymphocytes. PloS Pathog. 2 (7), e70. 10.1371/journal.ppat.0020070 16839201 PMC1500807

[B64] Rappocciolo G. Hensler H. R. Jais M. Reinhart T. A. Pegu A. Jenkins F. J. (2008). Human Herpesvirus 8 Infects and Replicates in Primary Cultures of Activated B Lymphocytes through DC-SIGN. J. Virol. 82 (10), 4793–4806. 10.1128/JVI.01587-07 18337571 PMC2346758

[B65] Rivas C. Thlick A. E. Parravicini C. Moore P. S. Chang Y. (2001). Kaposi’s Sarcoma-Associated Herpesvirus LANA2 Is a B-Cell-Specific Latent Viral Protein That Inhibits P53. J. Virol. 75 (1), 429–438. 10.1128/JVI.75.1.429-438.2001 11119611 PMC113935

[B66] Schmidt K. Wies E. Neipel F. (2011). Kaposi’s Sarcoma-Associated Herpesvirus Viral Interferon Regulatory Factor 3 Inhibits Gamma Interferon and Major Histocompatibility Complex Class II Expression. J. Virol. 85 (9), 4530–4537. 10.1128/JVI.02123-10 21345951 PMC3126280

[B67] Song S.-N. J. Tomosugi N. Kawabata H. Ishikawa T. Nishikawa T. Yoshizaki K. (2010). Down-Regulation of Hepcidin Resulting from Long-Term Treatment with an Anti-IL-6 Receptor Antibody (Tocilizumab) Improves Anemia of Inflammation in Multicentric Castleman Disease. Blood 116 (18), 3627–3634. 10.1182/blood-2010-03-271791 20644113

[B68] Sun C. Guo Y. Zhou W. Xia C. Xing X. Chen J. (2020). P300 Promotes Cell Proliferation through Suppressing Kaposi’s Sarcoma-Associated Herpesvirus (KSHV) Reactivation in the Infected B-Lymphoma Cells. Virus Res. 286 (September):198066. 10.1016/j.virusres.2020.198066 32553609

[B69] Totonchy J. Osborn J. M. Chadburn A. Nabiee R. Argueta L. Mikita G. (2018). KSHV Induces Immunoglobulin Rearrangements in Mature B Lymphocytes. PloS Pathog. 14 (4), e1006967. 10.1371/journal.ppat.1006967 29659614 PMC5919685

[B70] Ueda K. (2018). KSHV Genome Replication and Maintenance in Latency. Adv. Exp. Med. Biol. 1045, 299–320. 10.1007/978-981-10-7230-7_14 29896673

[B71] Uldrick T. S. Wang V. O’Mahony D. Aleman K. Wyvill K. M. Marshall V. (2010). An Interleukin-6-Related Systemic Inflammatory Syndrome in Patients Co-Infected with Kaposi Sarcoma-Associated Herpesvirus and HIV but without Multicentric Castleman Disease. Clin. Infect. Dis. 51 (3), 350–358. 10.1086/654798 20583924 PMC2946207

[B72] Wei X. Bai L. Dong L. Liu H. Xing P. Zhou Z. (2019). NCOA2 Promotes Lytic Reactivation of Kaposi’s Sarcoma-Associated Herpesvirus by Enhancing the Expression of the Master Switch Protein RTA. PloS Pathog. 15 (11), e1008160. 10.1371/journal.ppat.1008160 31751430 PMC6894885

[B73] Wies E. Mori Y. Hahn A. Kremmer E. Stürzl M. Fleckenstein B. (2008). The Viral Interferon-Regulatory Factor-3 Is Required for the Survival of KSHV-Infected Primary Effusion Lymphoma Cells. Blood 111 (1), 320–327. 10.1182/blood-2007-05-092288 17890449

[B74] Wu F. Y. Tang Q.-Q. Chen H. ApRhys C. Farrell C. Chen J. (2002). Lytic Replication-Associated Protein (RAP) Encoded by Kaposi Sarcoma-Associated Herpesvirus Causes P21CIP-1-Mediated G1 Cell Cycle Arrest through CCAAT/Enhancer-Binding Protein-Alpha. Proc. Natl. Acad. Sci. U.S.A. 99 (16), 10683–10688. 10.1073/pnas.162352299 12145325 PMC125013

[B75] Zhao Y. Ye X. Shehata M. Dunker W. Xie Z. Karijolich J. (2020). The RNA Quality Control Pathway Nonsense-Mediated MRNA Decay Targets Cellular and Viral RNAs to Restrict KSHV. Nat. Commun. 11 (1), 3345. 10.1038/s41467-020-17151-2 32620802 PMC7334219

